# Memoir of the Late James Hope, M. D.

**Published:** 1849-07

**Authors:** 


### ECLECTIC DEPARTMENT,
### AND SPIRIT OF THE MEDICAL PERIODICAL PRESS.

*Memoir of the late James Hope, M. D.*, Physician to St. George's Hospital, Author of "Diseases of the Heart," &c., &c., by MRS. HOPE. Edited by KLEIN GRANT, M. D. 4th Edition. London. 1848.

We have undertaken to prepare a review of the Life of Dr. Hope, from a conviction that it will greatly subserve the cause of medical education and of medical reform, which at this time so largely engages the attention of the community. The career of a distinguished professional man is interesting to those of his own profession, by presenting scenes with which they are familiar, objects which they have themselves pursued, difficulties with which they have had themselves to contend. A Medical Biography, therefore, if faithful, and well detailed, opens up a new scene to the mind of the reader, and commends itself to his reason, by the extensive materials it affords for thinkers, while it gratifies his curiosity by its freshness.

There are other circumstances which give peculiar interest to the memoir of this individual.

The subject attained great eminence, and large practice, at an age when most physicians are only beginning to be heard of. His success was not owing to the patronage of any great man, nor to any of those fortunate accidents which have occasionally brought physicians suddenly into notice. He was indebted solely to his own talents, his active humanity, the weight of moral character, and the force of industry, for his rapid elevation. These circumstances render the details of his life an encouraging example to the young physician—for, unhappily, the qualifications above mentioned are not the invariable, perhaps the truth will oblige us to confess, not the most frequent avenues to success in practice.

In one respect, the memoir possesses a more than usual degree of utility, since the circumstances under which it was written, enable us to trace, almost from infancy, the development of those powers and peculiarities, the exercise of which has led to such fine results.

It is the want of similar information that often renders the biography of individuals, however distinguished in literature, science or art, meagre and unprofitable—and it is only by shewing how the intellectual machine has been framed, and how it has worked in the production of the results we admire, that such memoirs can possess either interest or utility.

Dr. James Hope, the subject of this memoir, was born at Stockport, in the County of Cheshire, England, on the 23d of Feb., 1801, being the tenth child of a family of twelve children. His father, Thomas Hope, soon after the birth of his son, took up his residence at Presbury Hall in Cheshire, a venerable mansion, which was the fondly cherished scene of Dr. Hope's earliest associations, and boyish recollections.

Here, amidst the active sports and hazardous adventures which boys can only enjoy in the country, his character received that best of education which nature has prepared, both for the philosopher and the man of the world, and acquired the healthy force and activity which fitted him for the arduous situations in which he afterwards shone.

At the age of six or seven he was conducted to a school of some celebrity in the neighborhood. Here he seems to have acquired little more than the art of penmanship and of drawing maps, which he executed with singular beauty and correctness.

He early exhibited much avidity for reading and general information, his reading not being confined to books adapted to his age, or, as it was supposed, his capacity. When only eight years old, for example, his father found him intently perusing Milton's Paradise Lost, and, having chid him sharply for poring over what he could not understand, took the book from him. The boy could not comprehend the reason of this reproof, as he felt himself much interested in the story, and at length after many efforts, prevailed upon his father to allow him to proceed with it.

But his eagerness for knowledge, at nearly the same period, was more strikingly shown in the delight he experienced in reading Parke's Chemical Catechism, which also happened to fall in his way. He was quite fascinated with the science, and soon began to perform many of the experiments described in the book.

In these evidences of intellectual advancement he was strongly urged on by his father, who discovering rare talents in his son, was in the habit of stimulating him to the greatest exertion, and, by firing his youthful ambition powerfully urging him forward.

At ten years, he was removed to the grammar school of Knutsford, where he learned the rudiments of the classics, made great proficiency in arithmetic, and acquired the elements of geometry.

Having passed two years at that school, he was placed under the care of the Rev. G. S. Weideman, who received a few boys to prepare for public schools and Universities. Dr. W. led his pupil over a wide range of classics, including many of the higher ones. He did not restrict himself to classical instruction, as was too commonly the case at that period; but besides a due attention to mathematics, his plan embraced the elements of a philosophic and elegant education. By a well managed system of catechisms, this able preceptor familiarized his pupils with the first principles of natural philosophy and chemistry, as well as with the outlines of ancient and modern history and belles lettres.

By a critical analysis of English essays, he imbued the minds of his pupils with a knowledge of the general principles of style and taste. Under this system James Hope acquired the art of elegant English composition, became familiar with English classics, and imbibed the sentiments of the authors selected. To the two years thus spent, he was accustomed to look back with the highest satisfaction, as to a period in which he gained much general and diversified knowledge, and that expanse of intellect which rendered all his subsequent labors comparatively easy.

At the age of fourteen years he was placed at the Macclesfield Grammar School, under the care of Dr. Davies. This prince of pedagogues, as his pupils liked to call him, was an admirable critical classic, and a most successful disciplinarian. At this school he remained until he was seventeen or eighteen years of age, having read through nearly all the higher classics, Juvenal and Persius, most of the Greek plays, Thucydides, Pindar, &c.

His ambition was at this time to become a graduate of Oxford or Cambridge, in which reasonable desire he was opposed by his father, and after vain efforts to follow his inclinations, without success, he finally determined to qualify himself as a physician.

In 1820 he commenced his studies in Edinburgh, and his first year was devoted to anatomy, though the pursuit was highly uncongenial to his feelings. Instead of inquiring within himself what was agreeable or disagreeable, he calmly regarded the medical profession as that to which his destiny was linked, as the sphere in which all his bright and ambitious dreams were to be realized. He knew that habit is one of the most powerful agents on the mind of man, and he relied upon it for overcoming his feelings of disgust.

He took Dr. Baillie, at this time at the head of his profession, as the model which he proposed for his imitation. He soon discovered that this eminent physician owed much of his justly acquired celebrity to the knowledge which he derived from the study of morbid anatomy, and his own judgment enabled him to perceive that no man can hope to make important discoveries in medicine unless he is intimately acquainted with the structure and functions of the human body. The expression which he used many years after, in regard to this subject, was "that a physician, in looking at his patient, ought in imagination to turn him inside out."

Regarding this subject with the utmost antipathy, he determined to concentrate all his powers on the most essential, the least agreeable part of his studies. In so doing, he gave a striking proof of the strength of his moral character, and the result is an encouraging illustration of the fact, that a vigorous cultivation of the intellectual and moral powers, followed by a determined concentration of those powers on one object or class of objects, is almost invariably crowned with ultimate success.

He prosecuted his anatomical studies, particularly with a view to pathology, not as "mere morbid anatomy, the science of the dissecting room," but in reference to the previous symptoms—and in this, as in all other studies, he cultivated science purely as the foundation of practical knowledge, and in subserviency to it.

In 1822, he became a member of the Royal Medical Society of Edinburgh, and his connection with this Society occasioned an incident which first turned his thoughts to the selection of diseases of the heart as the subject of his chief investigation. Each member of this Society is required to write a paper, which is made the subject of an evening's debate. Dr. Hope wrote one on diseases of the heart. It was highly applauded, and a wish expressed that it might at a future day be expanded into a book. This circumstance first led him to give the subject particular attention, and on this foundation, subsequently enlarged, he constructed his valuable treatise on this subject.

In 1824 he was elected House Physician and House Surgeon to the Edinburg Infirmary. He was often heard to observe that the two years spent in the Edinburgh Infirmary were the most valuable of his life, as he literally lived at the bed side of his patients, where his sphere of observation was unlimited. In his election was exhibited a rare instance of one so young placed in charge of an important situation. A rule of the institution was also set aside, which required that the person holding the office of House Physician should have graduated. This did not take place until a year after, 1825.

The subject of his inaugural thesis was *aneurism of the aorta*, which had been declared by Lawrence to be impossible to make out. In the Edinburgh Infirmary, Dr. Hope had paid particular attention to these cases, and, as if in anticipation of his future discoveries, had already succeeded in doing that which had baffled Laennec. His motto was a quotation from his favorite Rambler, which from its soundness, and as an incitement, we will quote.

"All the performances of human art, at which we look with praise or wonder, are instances of the resistless force of perseverance. It is by this that the quarry becomes a pyramid, and that distant countries are united with canals. If a man was to compare the effort of a single stroke of the pickaxe, or of one impression of the spade, with the general design and last result, he would be overwhelmed by the sense of their disproportion; yet those petty operations incessantly continued, in time, surmount the greatest difficulties, and mountains are leveled, and oceans bounded by the slender force of human beings." *Rambler*, p. 43.

His taste for the fine arts was no less remarkable, and he attained conderable proficiency in music and painting. He had a fine taste and most accurate ear. This accurate ear was one reason of his excelling in auscultation, as this gave him a fine perception of the distinction of allied sounds. It is a striking fact, that the number of the best auscultators, including Laennec, who excelled on the violincello, have been musical amateurs. Whereas persons who cannot distinguish one tune from another, are comparatively bad auscultators.

Of painting, he was a still more devoted and successful follower. The great value of this art to the physician is too little considered, since, by means of it, he is enabled to delineate morbid productions. By steady perseverance he accumulated a considerable collection, and in the space of ten years he collected to the number of 300 or 400, comprising examples of almost every change of structure. In short, his motto was "age quod agis," and whether in the play ground, the school room, the hospital, the midnight study, or in society, he acted up to its spirit, seeming for the time to have no object beyond that in which he was engaged.

In 1826, he went to London for the purpose of studying surgery, and afterwards to Paris to acquire general knowledge, rather than to devote any special attention to his profession; when, after spending eighteen months there, he made the tour of Switzerland and Italy, and returning to England prepared to settle in London.

We come to the conclusion of James Hope's preliminary studies, and are now to witness the importance of steady habits and systemrtic training in modelling the future man. The field he selects is London, where every action is exposed to jealous and able scrutiny, and to which field he seemed prompted by a consciousness of his own talents, and by a proud ambition, which made him scorn success in *every* field, except that where he should have to compete with the highest order of talent.

In London, he stands alone, without any meretricious aid, and to his professional merits could he look alone for success. He entered London with but a single acquaintance, and the course he determines to pursue, is, with great diligence and prudence, to make himself known by his writings, by lectures, and especially by attaching himself to a hospital, with the hope of being its physician at a future day. To pass through this ordeal in London is indeed a test of talent, and it may be said that no man gains reputation in this way, and retains it, without deserving it. He assigns to himself the execution of the two works he had long planned; viz: a Treatise on Diseases of the Heart, and a complete work on Morbid Anatomy, illustrated by plates.

The execution of the first undertaking was not so easy, or so certain. This subject was then little understood, and although he had unravelled some of its intricacies, yet so much still remained uncertain and inexplicable, that he often doubted whether he should succeed in his object.. He determined not to publish until he should be fully competent to do so. To this end it was essential that he should continue his studies at some large hospital, and he selected St. George's as being the one to which he hoped one day to be physician. It was upon excellence in his profession that his efforts were concentrated to secure an enviable practice; and his opinions upon the several expedients resorted to to secure this end may with great propriety be noticed. He did not seek to form connexions as an introduction into practice, nor did he give himself much trouble about the cultivation of general acquaintance, except as a means of recreation, believing that much precious time was thus sacrificed, which might be better and more wisely spent in the furtherance of strictly professional objects. When a man becomes known as an agreeable man in society, as a musical performer, as an artist, as an adept in general science, in fact as any thing but a professional man, he loses his chance of securing a patient in almost the same ratio as he gains popularity. This was often noticed by him, and he remarked, not only that his friends and acquaintances were the last to discover his professional merits; but that, even when converted into patients, he had much less influence with them than with those whom he had first known as patients, and who were afterwards changed into friends.

Again, it is very generally believed that a physician should marry early, and that his practice will be benefitted by his assuming the character of a married man. Dr. Hope at one time participated in this notion, but his own experience so completely changed his opinion, that he was in the habit of warning his young friends against being led into a similar error. He rather advised, if they were influenced by considerations of policy alone, to defer forming this connection until an increased practice might enable them to do it with more advantage. He confessed that before his marriage he had believed the professions of some, who, while regretting that they could not employ an unmarried man, had given him to understand that the change of his state would make a corresponding change in their conduct. He found that this event made no addition to his practice, and that the hopes held out to him were fallacious.

Again, his opinion as to the giving of dinners to apothecaries and other members of the profession, in hopes of securing their professional assistance. This he considered a low device, from which his good taste no less than his pride revolted. From the first he set his face against it, and he believed it to be an equal loss of time, trouble and money. The persons thus invited at once see through the motive—they make a favor of accepting the invitation, praise the dinner, laugh at the host, and go away determined on following their own interest or inclination, uninfluenced by any recollection of the interested hospitality.

He, finally, often spoke of the imprudence of a man commencing his career in style, or setting up his carriage at too early a period—of course a certain respectability of appearance must be maintained from the first, and after some time the removal to a handsome house, and the getting up of a handsome carriage may be beneficial, as giving an impression of professional success. But if these are assumed at an early period, when it must be evident to all that they are supported from private, not professional resources, the owner loses all benefit of them at the time, and the power of resorting to them when he is legitimately entitled to do so.

Having settled himself in London, and become a pupil of St. George's Hospital, his entire thoughts were given to professional subjects, and among these was the completion of his works on the Diseases of the Heart and Morbid Anatomy.

It has been already mentioned in what manner his attention was first directed to the former subject. Other reasons operated with him for the consecration of his powers on this point. One was, that being a wide subject, researches in it were more likely to be useful than similar care devoted to the individual branches of any more explored branch of medical science: and secondly, that having been left in an unsatisfactory state by Laennec, there was more room for investigation. He continued his experiments on Diseases of the Heart, but without arriving at any satisfactory conclusions. At length he devised his ingenious experiments on the ass, and these, by enabling him to satisfy his mind as to the cause of the first sound of the heart, suddenly dissipated the greater part of the difficulties with which he had been contending.

As the subject of the *sounds of the heart* may be considered as possessing much special interest, we will endeavor to state the objects and results of these experiments, although we have on a former occasion, gone fully into the subject.

The action of the heart gives rise to certain sounds. If the ear be applied to the cardiac region of a healthy person, two successive sounds will be heard, then a brief interval of silence, then the repetition of the two sounds, then another interval, and so on, in a series which is perfectly regular and susceptible, indeed, of being represented by musical notation. The two sounds heard at each pulsation of the heart differ from each other both in kind and duration. The *first* sound is grave, louder at its commencement than at its termination, and seems to be suddenly interrupted by the *second sound*, which is short and acute, and has been compared to the flap of the valve of a pair of bellows. The first sound is synchronous with the impulse of the heart against the ribs, and with the pulse in the arteries nearest the heart. The existence of these sounds in the state of health, and the important indications afforded by the changes which they undergo in various disaases of the heart, had been established by Laennec, and were already familiar to those practised in ausculation.

The causes of these sounds. Laennec had given an opinion which for some time remained undisputed, viz: that the first sound was occasioned by the contraction of the ventricle, and the second by that of the auricle.

This explanation for a time remained uncontroverted, until Mr. Turner showed that the second sound could not depend on the contraction of the auricle, because the contraction of this cavity, in reality precedes that of the ventricle.

Dr. Hope's first experiments on the ass were made in August, 1830. The animal was stunnned by a blow on the head with a hammer, so as to render it insensible to pain, and the action of the heart was sustained by the maintenance of artificial respiration. The chest and pericardium being then opened, the central organ of the circulation was exposed to view, pulsating as in health. Dr. Hope's general conclusions from these experiments were, that the first sound is connected with the *contraction of the ventricle*, and the *second sound with the dilatation of the ventricle*—but the immediate causes of the sounds yet remained to be determined.

In three series of experiments made in 1835 on asses poisoned with woorara, Dr. Hope obtained certain results, which he deemed conclusive as to the causes of both sounds. From these he inferred that the first sound was compound, consisting 1st, of the valvular sound, 2nd, the sound of extension, and 3rd, of a prolongation of the sound by the muscular contraction; and that the second was produced by the closure of the sigmoid valves exclusively.

A large proportion of the obscurities that enveloped this subject being now removed, Dr. Hope no longer hesitated about publishing. He accordingly set about the work, and he wrote with such diligence that he completed in one year an octavo volume of 600 pages. This work met with the most favorable reception from all the principal reviews. It was hailed with a high degree of satisfaction, and his analysis of the sounds of the heart was pronounced to be the only great accession which the science of ausculation had received since the death of its memorable author, Laennec.* It was translated into German, passed rapidly through several editions in America, and found its way even into Italy.

In 1831 he was elected senior Physician to the St. Marylebone Infirmary, with a charge of ninety beds. In 1834 he turned his attention to the publication of his work on Morbid Anatomy. Delivered lectures on the Diseases of the Chest in the autumn of 1832, at his own house, with a regular attendance of from 30 to 40—mostly of practitioners.

*Of his character and habits of mind.*—In conversation he was full of interesting thought and information, and his manners were indicative of a peculiarly amiable and gentle disposition. He possessed the remarkable

---

* In no part of practical medicine has the progress of improvement been so good within the same period. For this we are largely indebted to Dr. Hope.—*American Journal of* 1842.

power of concentrating his mind at once on any subject to which he turned his attention.

His mind was always in a high state of activity, and when not engaged by any immediate object, seemed to be engrossed with subjects relating to his profession.

This was one great secret of success, as it is the great secret of the success of almost all those who have gained eminence in any department of science. Sir Isaac Newton, when asked how he made his discoveries, answered, "by always thinking about them," and at another time declared that "if he had done any thing, it was due to nothing but industrious and patient thought." Dr. Hope was so strongly impressed with this idea, that he often used to say that natural genius will do very little for a man without hard labor—and that almost all men who have distinguished themselves in literature or science, have been of diligent study.

*Of his practice.*—The first year after his settling in London, he had more practice than he had anticipated, and than generally falls to the lot a young physician. At the close of the year, Dr. Holland kindly inquired how he was getting on. Dr. Hope answered in cheerful terms, and mentioned how much he had made. "It does not signify," answered Dr. Holland. "how much or how little you have made, but what connexions have you formed, and what hold are you gaining in your patients' confidence." Dr. Hope saw the force of the observation, and perceived what was to be the aim of his conduct towards his patients, and the criterion of his success.

His practice diminished, and the third year of his residence in London, he made a smaller sum than at any other period. From the publication of the Treatise on the Diseases of the heart, in Nov., 1831, we may date the commencement of his practice. From this period it never fluctuated, but rapidly and permanently increased until he left town a few weeks before his death. His career terminated at the age of forty, and he was then making about £4000 a year.

In the spring of 1836, he accepted the situation of Lecturer on the Practice of Physic at the Aldersgate school of Medicine.

*His qualifications as a public Lecturer* were those of the highest order. He possessed all the highest attributes of a good professor. The analysis and division of his subjects, were clear, comprehensive and precise. During the entire series, all the powers of his mind were brought into action, and the immense mass of facts and observations collected by himself, were presented to his class, and placed in luminous apposition with all the leading opinions of the day.

He was gifted with a singularly pleasing elegance of expression which gave him a peculiar aptitude for tuition—and with all these qualities, he conjoined a generosity and amiability of disposition which won for him the collective admiration and affections of his pupils.

*His success as a Lecturer* was so great that he was subsequently offered the Lectureship of the Practice of Physic at many of the principal Medical schools which are unattached to hospitals. Of the labor he underwent to qualify himself for the situation of Professor, some idea may be formed from the following :—He says, "for the purpose of collecting materials and qualifying myself, I resided two years in the Edindurgh Infirmary as House Physician and Surgeon, and two more I spent in the Hospitals of

Paris, Heidelberg, Bologna, Florence and Rome. I have treated from 12 to 15000 hospital cases in this country, and seen nearly 3,000 post mortem examinations—made 5 or 600 drawings, and written 30 volumes of cases. Having done much of this specially for the purpose of lecturing, I do not like that my labor should be in vain."

*His success as a Practitioner* was based in a considerable degree upon his deep interest in his patients. There are not a few who can testify that his deportment in the sick chamber was but a faithful picture of such feelings. His extreme kindness, and the gentle tenderness of his manner, seldom failed to ensure him the affection of his patients, whether rich or poor, and some of his best personal friends date the commencement of that friendship from their own illness, or that of a relation.

In May, 1836, when he had begun to find his duties at St. George's Hospital too laborious, he had a slight cough and pain in his side, which yielded immediately to a blister, and he considered himself entirely re-established.

In the spring of 1837 he had an attack of Influenza, which settled on his chest, and from this period he had never been free from a slight hacking cough.

It was an established maxim of his, that a cough, however slight, should not be neglected; and therefore, he paid from the first, the greatest attention to the removal of his own. His health improved or deteriorated in exact proportion as his engagements were more or less arduous. He consulted Sir James Clark, who examined his chest, and forming an unfavorable opinion, recommended his going abroad. It not being in his power to do so, he advised that he should subject himself to as little fatigue as possible.

On the 19th of June he was attacked with spitting of blood, consequent upon much agitation and excitement, which he encountered upon the occasion of his election as physician to St. George's Hospital. From this time he dated the final breaking up of his health, which thenceforth progressively and rapidly declined.

As a parent, he was affectionate, tempering affection with judgment, and as he said, "loving his child too well to spoil him."

On the 22nd of December, 1839, he was attacked with pleurisy on the left side of his chest. For a few days he struggled against the disease, and continued his practice, but on the 26th he was obliged to confine himself to bed. On the 2nd of January, Dr. Chambers saw him, and two days later Dr. Watson, and from the first, they took the most serious view of his case. The following week Dr. Latham was called in, and from this period till his death, he was attended by Drs. Latham and Watson, with a kindness, which, had he been their brother, could not have been exceeded, and which his family feel that they can never sufficiently acknowledge. He died, May 22nd, 1841.

*Of his Religious Life.*—His studies were of a grave character. Perceiving what unfathomable depths of unattainable knowledge lay before him, he arrived at that rare wisdom inculcated by the Grecian sage, a conviction of his own ignorance. He entertained a very humble sense of his attainments, and forgetting the way he had already gone, he eagerly pressed forward with the feeling, that he had scarcely begun the race.

He read deeply upon the subject of church history, and especially the history of the Church of England, to which he was strongly attached.

His knowledge was extensive and diversified, he was ignorant alone on light and frivolous literature, there was no use to be made of it, and it was for profit not for amusement he read.

It was a remarkable circumstance in Dr. Hope's religious history, that his religion seemed in the first place to have been founded on his intellect, and through the instrumentality of his fine reasoning powers his heart became interested.

He heard a religious discourse, examined it as dispassionately as we would have done a physiological fact, and being satisfied of the evidence on which it rested, he received it as an acknowledged truth, placed it in his mind, of which it henceforth formed a part. To have doubted a truth so established, would have appeared to him as irrational and absurd as to have attempted to disprove a mathematical demonstration. It was a point settled, and he would as soon have thought of doubting the circulation of the blood, or again examining its proofs, as of unsetling his mind by the reception of a specious argument against a religious truth, which had once been established to his complete satisfaction.

In his practice he avoided as much as possible engagements on Sunday. Although attendance on the sick on Sundays is sanctioned by the example of our Saviour, and may be regarded as a work of mercy, by no means inconsistent with the sacred character of the Sabbath, some distinction ought nevertheless to be made between cases requiring immediate attendance, and those which are not affected by the delay of a day. Also, many cases are seen at intervals of a day or two, or on alternate days, and in those arrangements may be made to prevent the visit from falling on Sunday.

These were points to which Dr. Hope paid great attention. If he was obliged to see a patient on the Sabbath, he considered it in the light of a duty appointed by the Master whom he served, and he cheerfully obeyed the call; but he carefully avoided all unnecessary engagements, which must be regarded not as works of mercy, but as the pursuit of personal and worldly gain. He also made his appointments for hours which did not interfere with his attendance at church. He always attended divine service once, and by stopping at any church near which his engagements might lie, he generally contrived to go again in the afternoon.

With habits thus trained, and mind properly disciplined, he could advert to his approaching end without wavering, and could make minute and deliberate preparations for his departure. He made preparations for death as he had done for any important step that he had taken during his life. His family could find no more appropriate manner of describing his conduct, through the seven months that he lingered, than that it resembled that of a man, who expecting to set off on a journey, puts every thing in order before his departure and makes arrangements to supply his absence. He drew his hopes and conclusions from the Bible alone, and from that source he derived the joyful belief that in another world his renewed faculties and purified nature would enable him to love God more singly, and to serve him more actively than he had hitherto been able to do.—*Charleston (S. C.) Medical Journal and Review.*

